# The Total Ionizing Dose Effects on Perovskite CsPbBr_3_ Semiconductor Detector

**DOI:** 10.3390/s23042017

**Published:** 2023-02-10

**Authors:** Wuying Ma, Linyue Liu, Haoming Qin, Runlong Gao, Baoping He, Shilong Gou, Yihui He, Xiaoping Ouyang

**Affiliations:** 1 School of Nuclear Science and Technology, Xi’an Jiaotong University, No. 28, Xianning West Road, Xi’an 710049, China; 2 State Key Laboratory of Intense Pulsed Radiation Simulation and Effect, Northwest Institute of Nuclear Technology, Xi’an 710024, China; 3 State Key Laboratory of Radiation Medicine and Protection, Collaborative Innovation Center of Radiological Medicine of Jiangsu Higher Education Institutions, and School for Radiological and Interdisciplinary Sciences (RAD-X), Soochow University, Suzhou 215123, China; 4 Sino-French Institute of Nuclear Engineering and Technology, Sun Yat-Sen University, Zhuhai 519082, China

**Keywords:** perovskite, total ionizing dose, CsPbBr_3_, detector, irradiation

## Abstract

Perovskite CsPbBr_3_ semiconductors exhibit unusually high defect tolerance leading to outstanding and unique optoelectronic properties, demonstrating strong potential for γ-radiation and X-ray detection at room temperature. However, the total dose effects of the perovskite CsPbBr_3_ must be considered when working in a long-term radiation environment. In this work, the Schottky type of perovskite CsPbBr_3_ detector was fabricated. Their electrical characteristics and γ-ray response were investigated before and after ^60^Co γ ray irradiation with 100 and 200 krad (Si) doses. The γ-ray response of the Schottky-type planar CsPbBr_3_ detector degrades significantly with the increase in total dose. At the total dose of 200 krad(Si), the spectral resolving ability to γ-ray response of the CsPbBr_3_ detector has disappeared. However, with annealing at room temperature for one week, the device’s performance was partially recovered. Therefore, these results indicate that the total dose effects strongly influence the detector performance of the perovskite CsPbBr_3_ semiconductor. Notably, it is concluded that the radiation-induced defects are not permanent, which could be mitigated even at room temperature. We believe this work could guide the development of perovskite detectors, especially under harsh radiation conditions.

## 1. Introduction

The accurate detection of γ-rays from radioactive sources in high resolution at room temperature is of significant interest for industrial, medical imaging, and space applications [1,2,3]. However, the γ-ray photons are in very low flux. Notably, most semiconductors are susceptible to even a small number of defects and impurities that invariably act as signal-killing carrier traps. The traditional γ-ray detector materials are very few and face many challenges. The emergence of inorganic perovskite CsPbBr_3_ with surprisingly good defect tolerance, which is very promising for high-resolution X-ray and γ-ray detection at room temperature, has attracted extensive attention [4,5,6,7,8,9,10,11,12,13,14,15]. Additionally, the CsPbBr_3_ detector has shown remarkable energy resolving capability under both X and γ rays, particularly achieving 3.2% (3.9 keV, FWHM) energy resolution for 122 keV ^57^Co γ-ray [10,16]. Furthermore, the lifetime of the hole in CsPbBr_3_ detector-grade single crystal was observed to be over 25 μs [16].

For an excellent detector, device stability in a radiation environment is also critical. In addition, electronic devices for space, scientific and medical applications must be tolerant of a wide range of total ionizing doses (TID). However, the spectral degradation of the perovskite CsPbBr_3_ detector working in high irradiation environments has not been investigated [17,18,19,20].

In this work, we report a first investigation of the spectral response variation of CsPbBr_3_ detectors under total dose irradiation. The CsPbBr_3_ detector was fabricated with the asymmetric electrode design, in which the γ-ray response of the Schottky-type planar CsPbBr_3_ detector was revealed under different total doses. Additionally, the effect of the irradiation damage was evaluated through annealing experiments. Therefore, it is concluded that this device can work stably under the total ionizing dose of 100 krad(Si). We believe that our work will significantly promote and provide guidance for further investigations on the application of halide perovskites.

## 2. Materials and Methods

### 2.1. CsPbBr3 Detector Fabrication

**Figure 1:**
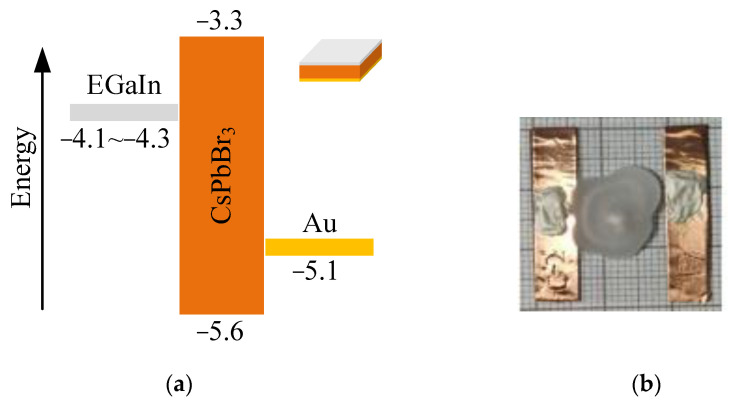
Architectural properties for CsPbBr_3_ detectors (**a**) Energy band diagrams for CsPbBr_3_ detector using asymmetrical electrode materials. (**b**) Top-view photograph of the CsPbBr_3_ detectors. As indicated in the insert, the dimension of the crystal is 3 × 3 mm^2^, where the thickness is 1 mm.

High-quality CsPbBr_3_ single crystals were grown from melt using the Bridgman method, as shown in Figure 1a. After mechanical polishing, the surfaces were deposited with the metal contact (Au and Ga/In), and the typical thickness of gold contact was ~70 nm. After electrode preparation, carbon paste was used to connect the metal electrode with the collection circuit for the front-end electronics. Finally, the test sample is sealed with wax. And the top-view photograph of the CsPbBr_3_ detectors was illustrated in Figure 1b. More information on the samples is detailed in our previous work [16].

### 2.2. Irradiation

The CsPbBr_3_ detectors were irradiated by γ-rays derived from the ^60^Co source (at the Northwest Institute of Nuclear Technology, Xi’an, China) and had an energy distribution centered typically around 1.25 MeV. The detector was unbiased, with all pins floating during ^60^Co γ radiation, and tested at a dose rate of 50.0 rad(Si)/s. The sample’s current-voltage (IV) characteristics were measured at the total ionizing doses of 100 and 200 krad(Si), respectively. The spectrum response of the sample was measured at the total ionizing doses of 100 and 200 krad(Si). Finally, the sample’s tested parameters were measured within two hours after each radiation step.

### 2.3. Measurements

**Figure 2:**
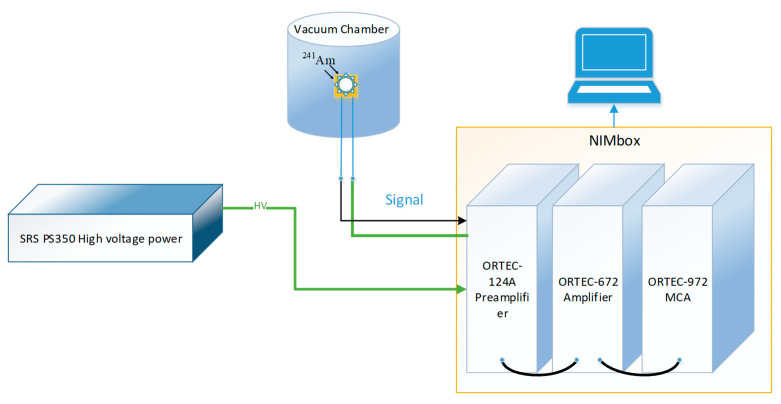
Schematic diagram of the γ-spectra measurement system.

The current-voltage (IV) characteristics of the CsPbBr_3_ detector were measured using a Keysight B1500A semiconductor device analyzer. The response was tested with a 59.5 keV ^241^Am γ-ray source. As shown in Figure 2, the CsPbBr_3_ detectors were positioned inside a vacuum chamber to detect γ-rays. The response signal induced in the CsPbBr_3_ detector was, in turn, transmitted to the following equipment through coaxial cables: an ORTEC-142A preamplifier, an ORTEC-672 amplifier (shaping time of 3 μs, gain of 1500 times), an ORTEC-ASPEC-927 multi-channel analyzer (MCA), and the MAESTRO software was used to acquire the γ-ray spectrally. An SRS PS350, a high-voltage supply, was used to provide the reverse bias to the CsPbBr_3_ detector through the preamplifier.

## 3. Results and Discussion

### 3.1. I-V and I-T Characteristics

**Figure 3:**
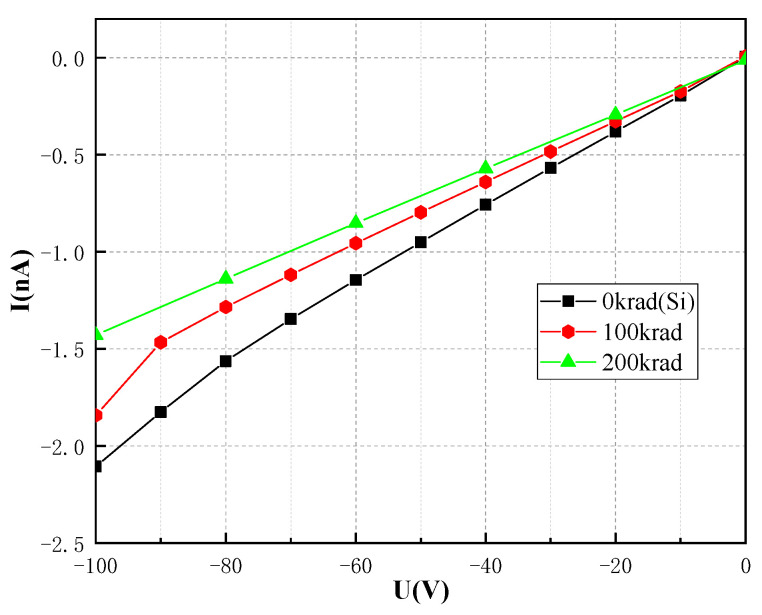
Typical dark I–V reverse characteristic curve of the CsPbBr_3_ detector.

As shown in Figure 3, The pristine I–V curve of the CsPbBr_3_ detector on the reverse side was compared with the reverse side I–V curves taken after the irradiation with the total dose of 100 krad and 200 krad, respectively. During the test, the sweep voltage was set from −100 V to 0 V, with 4 s intervals between each voltage step. As indicated in Figure 3, with the increase in total dose, there was a decrease in the dark current of the CsPbBr_3_ detector. Notably, the higher the bias voltage applied, the more the radiation effect will be.

**Figure 4:**
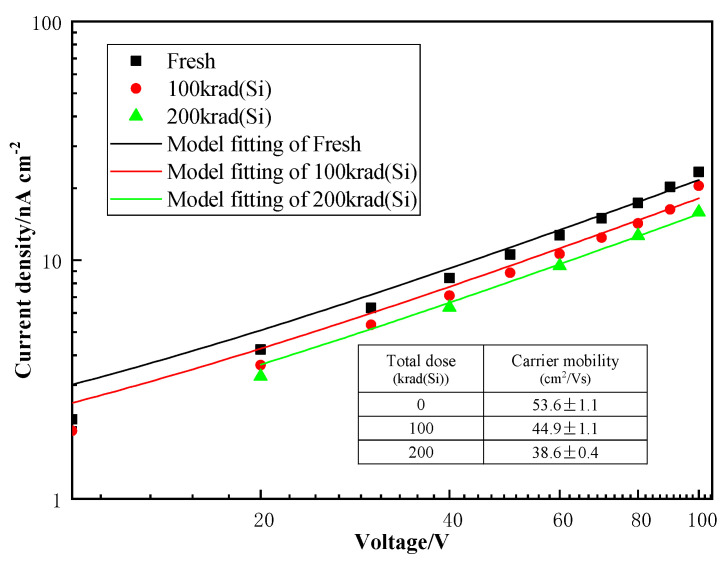
The J-V characteristics and its tendency fit according to the diffusion model.

The diffusion model has been utilized for analyzing the I–V characteristics of the CsPbBr_3_ detector [21,22,23,24,25]. In the diffusion model, the current density J under the reverse bias V in a metal-semiconductor Schottky device can be described as:

(1) $$J=\frac{q^{2}\mu N_{c}N_{i}}{\varepsilon }\left[\frac{\varepsilon }{qN_{i}d}(V+V_{in})+\frac{d}{2}\right](1-e^{-qV/kT})e^{-q\varphi /kT}$$

where *q* is the electron charge, *μ* is the carrier mobility, *N_c_* is the effective density of states in the conduction band, *N_i_* is the concentration of the ionized donor/acceptor centers, *ε* is the electrical permittivity in the crystal, *d* is the thickness of the device, *V_in_* is the built-in internal electric field, *k* is Boltzmann’s constant, *T* is temperature, and *ϕ* is Schottky barrier [24]. Accordingly, the J–V curve under the reverse bias of a CsPbBr_3_ device was fitted according to the above J–V equation, as plotted in Figure 4.

Accordingly, the J-V curve under the reverse bias of an MSM device was fitted according to the above J-V equation, as plotted in Figure 2. The J–V curve in the measured range followed the tendency predicted by the diffusion model. Before irradiation, the Schottky barrier extracted was 0.72 eV, while u is 53.6 cm^2^/Vs. Following irradiation, the Schottky barrier was not changed, while the μ was dropped with the total dose, and 44.9 cm^2^/Vs for 100 krad(Si) 38.6 cm^2^/Vs for 200 krad(Si). Thus, we considered that the radiation induced defects in the CsPbBr3 detector and is the main reason for the decrease in mobility.

**Figure 5:**
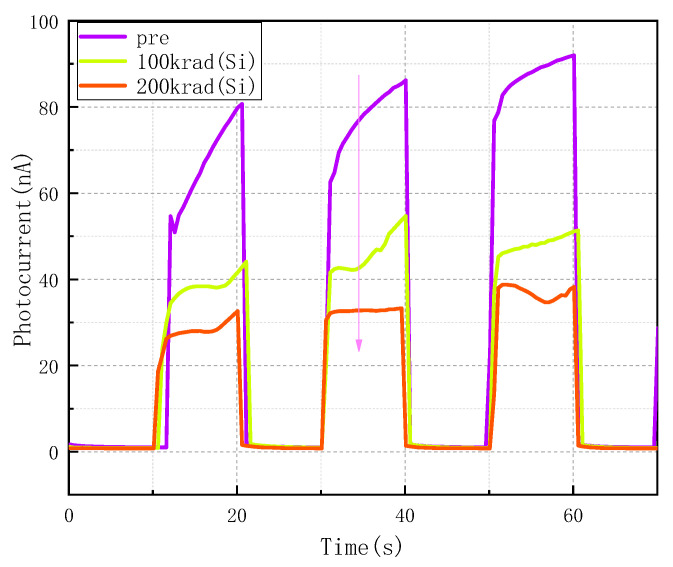
Typical I–T characteristic curve of a Schottky type CsPbBr_3_ detector after radiation. The light source is a 40 W fluorescent lamp installed in the dark box and switched ON and OFF at 10-s intervals.

The total ionizing dose impacted the photoelectric response of the CsPbBr_3_ detector. Figure 5 shows the time-dependent currents under the light pulses and 50 V bias. The dark current was about 0.7–1.9 nA. Before radiation, the photocurrent was around 85 nA, and the ON-OFF ratio was about 80. Then, with the increase in total dose, the photocurrent and the ON-OFF ratio dropped. For the total dose of 200 krad(Si), the photocurrent was around 32 nA, and the ON-OFF ratio was reduced to 35. Therefore, this finding indicates that electrical-active defects were likely formed after total irradiation, resulting in the trapping of photo-generated carriers.

### 3.2. γ-ray Spectral Performance

**Figure 6:**
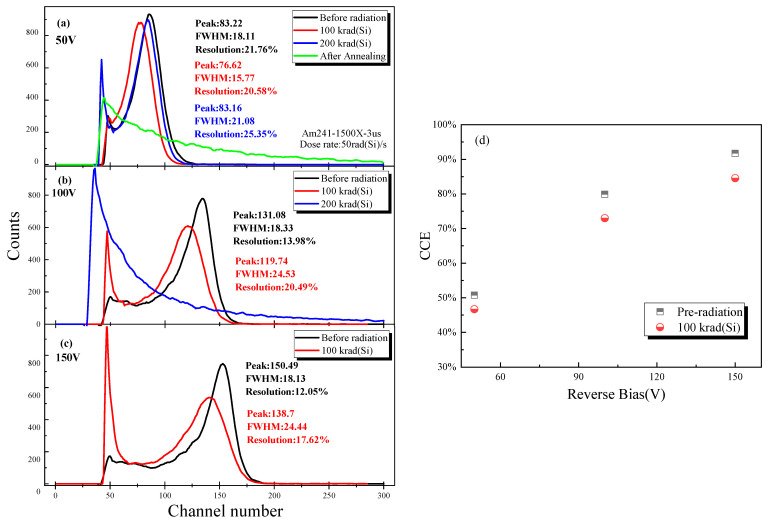
The γ ray spectra by the Schottky type CsPbBr_3_ detector under a 241Am γ-source with different applied bias voltage before and after radiation. (**a**) −50 V, (**b**) −100 V, (**c**) −150 V. (**d**) The calculated CCE is based on the spectra indicated in (**a**–**c**). The curve was smoothed by the Savitzky–Golay method.

The γ-ray spectral performance of the CsPbBr_3_ detector was also influenced by total ionizing dose radiation. The energy resolution is a critical characteristic for evaluating the ability of energy spectral measurement of the detectors. The detector’s energy resolution is usually defined as the ratio between the FWHM and the position of the peak centroid, which is a dimensionless fraction expressed as a percentage. Figure 6 shows the energy resolution of CsPbBr_3_ detectors to ^241^Am γ-ray with various bias voltages (−50 V, −100 V, and −150 V) before and after ^60^Co γ ray total dose irradiation. During the energy spectral test, the radiation-induced charge Q collection was on the Au cathode, while Ga/In anode was grounded, and the γ-ray irradiated from the anode. Figure 6a illustrates the energy spectra under applied voltage (−50 V) with a shaping time of 3 µs. Before radiation, the CsPbBr_3_ detector showed a well-resolved spectroscopic response with an energy resolution of 21.76% for 59.5 keV ^241^Am γ-ray. Then the energy resolution degraded to around 34.2% after γ-ray irradiation with a total dose of 100 krad(Si). At the same time, the spectral performance disappeared when the total dose increased to 200 krad(Si). After the radiation device had been annealed at room temperature for one week, the γ-ray spectral response was restored with an energy resolution of 25.35%. During the annealing test, the pins of the device were in floating bias and were placed at room temperature. Figure 6b shows that when the applied bias was −100 V, the TID effects on the spectral performance of the CsPbBr_3_ detector. Before irradiation, the CsPbBr_3_ detector showed a more well-resolved spectroscopic response with an energy resolution of 13.89%. After a cumulative total dose of 100 krad(Si), the energy resolution was 20.49%. When the total dose reached 200 krad, the spectral performance disappeared. After annealing, the device’s performance under this voltage bias has not been recovered.

The test result with −150 V bias voltage is demonstrated in Figure 6c. The higher the bias voltage applied the better spectral performance was achieved. However, under higher bias voltage, the influence of irradiation becomes more significant. As shown in Figure 6c, the energy resolution was 12.05% before irradiation and increased to 17.62% after irradiation with a total dose of 100 krad(Si). Similar to the test results under −100 V bias, there was no energy spectrum characteristic when the cumulative total dose was 200 krad(Si) and the test after annealing.

The charge collection efficiency (CCE) is also influenced by the TID effect, as shown in Figure 6d. The CCE of CsPbBr_3_ detectors can be calculated by using a Si detector as a reference sample, and CCE can be expressed as in Formula (2) [26,27,28].

(2) $$CCE=\frac{P_{CsPbBr_{3}}}{P_{Si}}\times \frac{G_{Si}}{G_{CsPbBr_{3}}}\times \frac{Ed_{CsPbBr_{3}}}{Ed_{Si}}\times \frac{E_{\alpha }}{E_{\gamma }}\times CCE_{Si}$$

The *P_Si_* and *P_CsPbBr3_* represent the channel numbers of the peak centroid measured by the CsPbBr_3_ detector and Si (697.52) detector, respectively. The *G_Si_* and *G_CsPbBr3_* represent the gain for CsPbBr_3_ (50) and Si (1500), respectively. The $E_{\alpha }$ and $E_{\gamma }$ and present the α-particle energy of ^239^Pu (5156 keV) and γ-ray energy of ^241^Am (59.59 keV), respectively. The *Ed_Si_* and *Ed_CsPbBr3_* represent the mean ionization energy for CsPbBr_3_ (5.3 eV) and Si (3.6 eV) [26], respectively. The *CCE_Si_* is the charge collection efficiency of the reference silicon detector (~100%). The CCE at 150 V bias of the pre-irradiated CsPbBr_3_ detector was 91.75% and decreased to 84.56% with a total dose of 100 krad (Si). The degradation of energy resolution and CCE can be ascribed to increased trapping centers under gamma radiation, which decrease the mean free path of holes and electrons.

### 3.3. Photoluminescence

**Figure 7:**
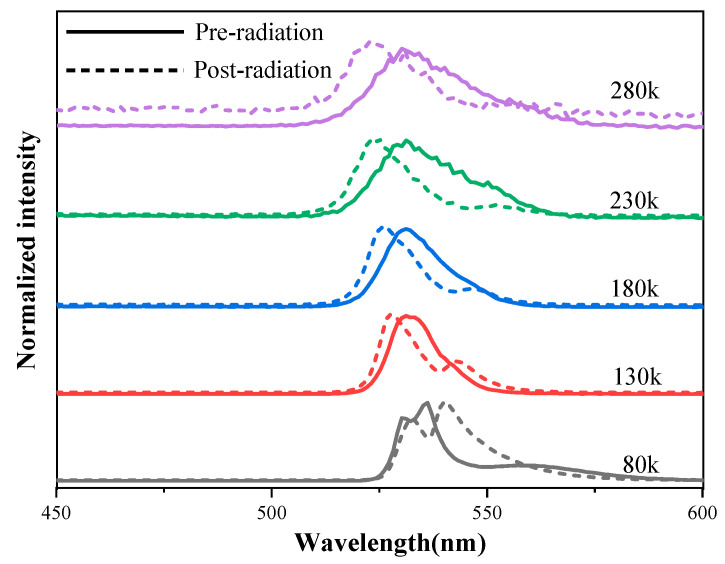
Temperature-dependent PL spectrum excited by a 365 nm laser beam, all normalized to clarify the peak shift.

PL spectroscopy was used to characterize the optical properties of the perovskite CsPbBr_3_ crystal. Figure 7 shows the PL spectrum of as-grown and ^60^Co γ-ray irradiated samples (the total dose = 165 rad(Si)), and the PL intensities at various temperatures were analyzed in detail. It is evident that the peak value of the PL spectrum has a red shift in the 80 K temperature test, and with the increase of test temperature, the peak value of the PL spectrum after irradiation appeared to have a blue shift. The possible reason for this phenomenon is the total ionizing dose irradiation changes the potential energy at the top of the valence band and at the bottom of the guide band of the halide material [26]. The origin of this difference needs to be further investigated.

## 4. Discussion

This work investigated the ^60^Co γ ionization radiation effects on the Schottky type CsPbBr3 detector at the dose rate of 50.0 rad(Si)/s. The ionization radiation has little effect on the I–V curve of the CsPbBr_3_ detector. With the increase in total dose, the dark current decreased slightly. The I–T character was impacted significantly after ionization radiation, and the pre-irradiated CsPbBr_3_ detector demonstrated a high ON-OFF ratio of 80. After 200 krad(Si) radiation, the ON-OFF ratio of the CsPbBr_3_ detector was reduced to 35. At 100 krad(Si), the energy spectral characteristics of the device were still maintained. However, when the total dose accumulated to 200 krad(Si), the spectral performance of the tested CsPbBr_3_ detector disappeared.

As shown in Table 1, with various bias voltages (−50, −100, and −150 V) during the γ-ray spectral test, there were different responses of energy resolution to irradiation ΔR = R1 − R2, where R1 represents the resolution of post-irradiation and R2 represents the resolution of pre-irradiation. When tested under extensive voltage conditions, the energy resolution caused by radiation after accumulating 100 krad(Si) was deduced at about 6%. Additionally, the performance did not recover within one week of room temperature annealing. Then, under low-voltage test conditions (50 V), the spectral resolution slightly increased by 1.1% after accumulating a total dose of 100 krad(Si). In addition, the main difference was that when tested under low voltage conditions, the energy spectral performance of the CsPbBr_3_ detector recovered within one week of annealing after irradiation. Additionally, the energy resolution after annealing degraded by about 4.77% compared to that obtained before irradiation. However, this recovery process was not observed when testing at high voltage. Finally, when the total dose was 200 krad(Si), the detector had no spectral characteristics tested under all bias conditions.

## 5. Conclusions

The ^60^Co γ ionizing radiation may induce electrically active defects in the bulk of the metal-semiconductor interface of the CsPbBr_3_ detector. Those defects will likely alter the detector’s performance. The difference in energy spectrum under different test bias voltage may also be due to the defect induced by ionizing radiation in the Schottky interface region. With the increase of the bias applied during the testing process, the Schottky barrier becomes wider, and the space charge region increases, leading to the decrease of carrier lifetime during the energy spectrum testing. Therefore, our future work will continue to study the radiation effects and damage mechanism of the CsPbBr_3_ detectors to meet the increasing demands for radiation detection in intense radiation fields.

## Figures and Tables

**Table 1:** Total dose response of the CsPbBr3 detectors under different test biases.

Bias Voltage (V)	△Energy Resolution		
	−50	−100	−150
100 krad(Si)	−1.18%	6.42%	5.57%
After annealing	4.77%	--	--

## Data Availability

Not applicable.
